# *In vitro* Prebiotic Effect of Bread-Making Process in Inflammatory Bowel Disease Microbiome

**DOI:** 10.3389/fmicb.2021.716307

**Published:** 2021-10-11

**Authors:** Aleix Lluansí, Marc Llirós, Lia Oliver, Anna Bahí, Núria Elias-Masiques, Marina Gonzalez, Patrícia Benejam, Emilio Cueva, Miquel Termes, Sara Ramió-Pujol, Marta Malagón, Joan Amoedo, Marta Serrano, David Busquets, Leyanira Torreabla, Miriam Sabat, Maria Buxó, Maria Cambra, Mariona Serra-Pagès, Sílvia Delgado-Aros, Liberado Jesús García-Gil, Isidre Elias, Xavier Aldeguer

**Affiliations:** ^1^Digestive Diseases and Microbiota Group, Institut d'Investigació Biomèdica de Girona, Salt, Spain; ^2^GoodGut S.L., Girona, Spain; ^3^Elias–Boulanger S.L., Vilassar de Mar, Spain; ^4^Digestive Service, Hospital Universitari de Girona Dr. Josep Trueta, Girona, Spain; ^5^Boehringer Ingelheim International GmbH, Ingelheim am Reim, Germany; ^6^Department of Biology, University of Girona, Girona, Spain

**Keywords:** gut microbiota, bread, sourdough, fermentation, dysbiosis, microbial ecology

## Abstract

Inflammatory bowel disease (IBD), including its two main categories (Crohn's disease and ulcerative colitis), has been linked both to gut microbiota and to diet. Bread is a daily food that has a potential capacity as a prebiotic. Our aim was to evaluate different bread-making processes and their effect on fecal colonic microbiota in IBD patients. The microbial composition of several sourdoughs and dough samples was analyzed by high-throughput sequencing of 16S and 18S rRNA genes. Three types of bread, which followed different bread-making processes, were *in vitro* digested and incubated with feces from IBD patients. Changes in gut microbiota were assessed by a quantitative polymerase chain reaction using specific bacterial sequence targets. Short-chain fatty acid production was also analyzed by gas chromatography. *Lactobacillus sanfranciscensis* was the dominant lactic acid bacteria species found in sourdough and bread doughs prepared using sourdough, whereas *Saccharomyces cerevisiae* was the most dominant yeast in all groups, especially in bread doughs before baking. Differences in microbial composition in raw bread doughs were more related to the type of dough and elaboration than to fermentation time lengths. The analysis of *in vitro* fecal incubations with bread conditions revealed an increase in most bacterial groups analyzed and short-chain fatty acid production, both in Crohn's disease and ulcerative colitis samples. Most remarkable increases in short-chain fatty acid production mirrored higher abundances of *Roseburia* species. The potential prebiotic properties observed were mainly obtained when using a high quantity of bread, regardless of bread type. Overall, this study highlights the bacterial dynamics within the bread-making process and the potential prebiotic effect in IBD patients.

## Introduction

Inflammatory bowel disease (IBD) is a group of immunomediated chronic inflammatory intestinal conditions, with Crohn disease (CD) and ulcerative colitis (UC) as main categories. They both have overlapping and distinct clinical and pathologic features (Silverberg et al., 2005; Bernstein et al., 2016), but typically CD can produce transmural inflammation and affect most commonly ileum and right-sided colon. In contrast, UC is typified by superficial colonic inflammation, shallow erosions, and ulcers, all of them limited to the colon (Bernstein et al., 2016). In general terms, IBDs evolve throughout stages of relapses (active phase) or remission (inactive phase), both of variable duration and intensity. The etiology of IBDs is incompletely understood. Researchers have proposed their origin as multifactorial, suggesting that environment, genetic factors, immunologic response, and gut microbiota are involved in their pathology (Eckburg et al., 2005; Loddo and Romano, 2015).

The role of the gut microbiota in the onset and perpetuation of intestinal inflammation in IBD has been systematically studied over the last decades (Eckburg et al., 2005; Flint, 2012; Cao et al., 2014). It is assumed that the gut microbiome has high relevance in health. Besides fundamental metabolic functions, it is clue to modulate the relative production of proinflammatory vs. anti-inflammatory signals received by the immune system (Flint, 2012). The gut microbiota has also been extensively linked to host immunity development and gut homeostasis maintenance (Sears, 2005). Moreover, it is well-established by studies performed both in fecal and mucosa-associated communities that CD patients have an altered microbiota, which differs from that found in patients with UC and both from healthy controls (Flint, 2012).

In IBD patients, a reduction of butyrate-producing species abundance was observed correlated with changes in the composition of fecal organic acids compared with healthy subjects (Nemoto et al., 2012). The importance of butyrate-producing species in gut microbiota relies on this compound's anti-inflammatory activity (Klampfer et al., 2003; Schwab et al., 2007). Butyrate has a crucial role in gut physiology, as it is an energy source for the intestinal mucosa and promotes the growth and differentiation of colonic epithelial cells (colonocytes) (Stein, 2000; Hamer et al., 2008; Topping and Clifton, 2018). Furthermore, intestinal organic acids, among which butyrate is an important one, have immunomodulatory effects on colonic inflammation and a reinforcing effect on the intestinal defense barrier (Stein, 2000; Cavaglieri et al., 2003; Hamer et al., 2008). Evidences pointed toward a critical role of butyrate-producing bacteria such as *Faecalibacterium prausnitzii, Roseburia* species, and *Subdoligranulum* species (Sokol et al., 2008; Louis and Flint, 2009; Lopez-siles et al., 2012). For instance, *F. prausnitzii* can account for up to 20% of fecal microbiota in healthy individuals (Lopez-Siles et al., 2015), and a reduced relative prevalence and abundance in gut diseases either in fecal or mucosal samples, in particular in IBD, have also been described (Sokol et al., 2009; Lopez-siles et al., 2012, 2014; Chatel et al., 2013). On the contrary, the higher contribution of microbes such as *Escherichia coli* to the gut microbiome can dramatically affect gut epithelial integrity, thus increasing epithelial barrier dysfunction and IBD physiopathology (Mirsepasi-lauridsen et al., 2016).

Current therapies on public health systems are based on attenuation of local inflammation in the colon and other parts of the digestive system. Modulation of colonic luminal ambient may play an essential role in treating and maintaining IBD remission (Bamba et al., 2002). Gut microbiota modulation can be obtained from several approaches, including probiotic and prebiotic supplements, diet, and correction of other factors that produce gut microbiota alterations (Scaldaferri et al., 2013).

Prebiotics are generally defined as non-digestible food ingredients, mainly carbohydrates that escape from digestion in the upper gastrointestinal tract and beneficially affect the host by selectively stimulating the growth and activity of one or a limited number of microbes in the colon and thus improve host health status (Gibson and Roberfroid, 1995). The bacterial metabolic routes involved in food digestion are strongly conditioned by the nutrients, particularly dietary fiber and complex polysaccharides. It is generally well-accepted that dietary fiber induces short-chain fatty acid (SCFA) production, mainly acetic, butyric, and propionic acids, which are essential nutrients for epithelial cells and gut microbiota (Sartor, 2008).

Bread is a universal food containing carbohydrates, B vitamins, minerals (phosphorus, magnesium, and calcium), and vegetable proteins, plus dietary fiber sources for our intestinal bacteria (Arias et al., 2017). The bread-making process, including dough composition and fermentation, impacts the final microbial and chemical composition of bread (Tamang et al., 2016). Traditional bread-making uses sourdough, a leavening agent traditionally obtained from a mixture of flour and water and spontaneously fermented by a complex microbiota dominated by lactic acid bacteria (LAB) and yeast population. Empirical data, either from animal models (Arias et al., 2017) or *in vitro* model studies using human stool samples (Costabile et al., 2014), indicate that traditional bread might positively impact gut microbiota, immunity gut responses, and systematic inflammation compared to industrial bread ingestion. Other evidence points toward an improvement of gut microbiota composition when using whole-grain flour for bread production (Costabile et al., 2008). Furthermore, the fermentation process seems to have significant consequences on the final fiber content and the bread's carbohydrate and protein complexity (Saa et al., 2017). Although abundant literature has dealt with the characterization of the mature sourdough microbiota (De Vuyst et al., 2002; Meroth et al., 2003; Scheirlinck et al., 2007; Minervini et al., 2012a), studies aimed at revealing the microbial dynamics leading from initial dough to mature sourdough or the microbial composition of raw dough at different fermentation times are scarce, limiting knowledge on dough prebiotic properties.

We aim to investigate bread consumption as a possible prebiotic therapy for IBD. To reach this target, we assessed the effect of different types of bread in terms of chemical composition, production, and fermentation on fecal microbiota from IBD patients. Besides, we performed high-throughput sequencing on sourdough and raw doughs used to elaborate these types of bread to monitor the microbial community dynamics during bread preparation.

## Materials and Methods

### Experimental Setup

#### Sourdough Elaboration

Sourdough was prepared as described below (proportions are based on weight and with respect to flour): A starter was generated by mixing equal weight parts of whole-grain wheat flour (70%, T110 flour, *Triticum dicoccoides*; Moulin de Colagne®, France) and water (30%) resting at room temperature (19−20°C) for 24 h, reaching final pH value of 6.79 and total titratable acidity (TTA) of 14.02 mL. Every day over a 5-day period, successive addition of equal parts of T110 flour and water (i.e., 1 kg) was performed reaching a final 10-kg mix. pH and TTA were followed up over all starter propagation period never exceeding 6.8 pH units and 10.50 mL of TTA. On the 5th day, the 10-kg mixture was combined with fresh *Thymus vulgaris* and *Rosmarinus officinalis* infusion, pasteurized ecological goat yogurt, green golden apple skin, *Syzygium aromaticum*, and *Cinnamomum verum* (following baker's own receipt) and a final addition of equal parts on weight (i.e., 1 kg) of T110 flour and water. The mix was left to rest at room temperature. On the 6th day, 108% of T110 flour was added to the previous mixture in order to dehydrate and saturate the mixture and preserve it over a 6-month period without losing qualities. The final started has a temperature of 34°C, pH value of 6.68, and TTA of 13.73 mL. The initial dough was prepared by mixing the starter (30%), water (50%) and whole-grain wheat flour pressed through stone mill (70%, T110 flour, *T. dicoccoides*; Moulin de Colagne®). Afterward, a procedure called feeding or back-slopping (where the initial mixture was used to ferment a new mixture of water and flour repeatedly every 12–24 h for 7 days) was performed to induce the microbial fermentation or propagation of sourdough. The resulting homogeneous mass was called sourdough or MMC. Samples were taken during the preparation of the sourdough after 0 h (mmc0), 48 h (mmc48), 96 h (mmc96), and 168 h (mmc168) of fermentation.

#### Raw Bread Doughs and Bread Preparation

Sourdough (wt/wt; 15% flour basis) was mixed with whole-grain wheat flour (*Triticum aestivum*) pressed with stone mill “Moulin de Coulagne,” tap water (wt/wt; 40% flour basis), dry baking yeast (*Saccharomyces cerevisiae*, wt/wt; <0.5% flour basis; Lesaffre (Hirondelle®), and salt (wt/wt; 1.1% flour basis; Guerande®) using a kneading machine (Sancassiano®) for up to 15 min. So far, we called this mixture Elias–Boulanger long-fermentation raw dough (eblfmc). Then, a double-stage fermentation process [i.e., dough maturation stage at 4–6°C (during either 44 or 68 h) and a dough development stage at 28°C for 4 h] was carried out under lactic–acetic fermentation conditions and 70% humidity in a fermentation chamber (Eurofours®). Samples were taken at 0 (eblfmc0), 48 (eblfmc48), and 72 h (eblfmc72) of fermentation from the above-described doughs. Finally, fermented doughs were baked at 200°C for 90 min in a refractory sole oven (Eurofours®). The 72-h fermented final baked product was called Elias–Boulanger long-fermentation bread 1 (eblfb1), whereas the one fermented 48 h was called Elias–Boulanger long-fermentation bread 2 (eblfb2). Final pH values for both breads were 4.4 pH units. Both breads had a slightly red crust, with a toasted wheat and malt aroma, and a marked sweet taste of cereals but differed in the humidity and acidity of the crumb, which was in both cases elastic.

A white bread, without using sourdough, was prepared in parallel as follows: refined wheat flour (wt/wt 57% flour basis, Farinera Corominas®), tap water (wt/wt 40% flour basis), salt (wt/wt 1.2% flour basis, Sal Costa®), yeast (*S. cerevisiae*, 0.010 g/kg of flour; Lesaffre (Hirondelle®), xanthan gum (0.005 g/kg of flour), wheat gluten (0.010 g/kg of flour; Uniplus®), enzymes (α-amylase, endoxylanasse, amyloglucosidanassa; Uniplus®), and additive components: emulsifier E471, antioxidant E-300 (Uniplus®), were mixed using a kneading machine (Sancassiano®) for up to 23 min. This initial mixture was called Elias–Boulanger industrial raw dough (ebindmc). Afterward, a fermentation process was carried out under fully controlled conditions for rapid fermentation processes at 28°C for 2 h under controlled conditions (70% humidity) in a controlled fermentation chamber (Eurofours®). Samples were taken at 0 (ebindmc0) and 2 h (ebindmc2) of fermentation. Then, samples were baked at 200°C for 90 min in an oven (Eurofours®). The resulting final product was named Elias–Boulanger industrial bread (ebindb).

### Microbial Diversity

Total genomic DNA was extracted from bread dough samples: MMC or sourdough after 0 h (*n* = 1), 48 h (*n* = 1), 96 h (*n* = 1), and 168 h (*n* = 1) of elaboration; eblfmc or Elias–Boulanger long-fermentation raw dough after 0 h (*n* = 5), 48 h (*n* = 3), and 72 h (*n* = 3) of fermentation; ebindmc or Elias–Boulanger industrial raw dough after 0 h (*n* = 3) and 2 h (*n* = 3) of fermentation. DNA extraction was performed by using the NucleoSpin Soil Kit (Macherey-Nagel GMbH& Co., Duren, Germany) following manufacturer instructions and eluted in 100 μL of Elution Buffer. Total genomic DNA was quantified using a Nanodrop ND-2000 UV-Vis spectrophotometer (Nanodrop, DE) and Qubit® (ThermoFisher Scientific®) measurements.

The V3–V4 region of the bacterial 16S rRNA gene and the V9 region of the eukaryotic 18S rRNA gene were amplified from each sample following common practices at external facilities (StarSEQ GmbH). Briefly, triplicate end-point polymerase chain reaction (PCR) reactions for V3–V4 and V9 regions were performed using previously described primers [V3–V4 16S rRNA gene region, 341f−806r (Klindworth et al., 2013); V9 18S rRNA gene region, Illumina_Euk_1391f–Illumina_EukBr_1510r (Amaral-Zettler et al., 2009)], and equimolarly pooled to reduce bias. Pooled libraries were gel purified and spiked with 8.5–10% PhiX before sequencing. Paired-end (2 × 300 base pairs) high-throughput DNA sequencing was carried out using the MiSeq platform (Illumina®). Obtained reads were processed with Cutadapt, DADA2 pipeline, and phyloseq R packages (Martin, 2011; McMurdie and Holmes, 2013; Callahan et al., 2016). Default settings used were for filtering and trimming. Built-in training models were used to learn error rates for the amplicon dataset. Identical sequencing reads were combined through DADA2's dereplication functionality, and the DADA2 sequence–variant inference algorithm was applied to each dataset. Subsequently, paired-end reads were merged, a sequence table was constructed, taxonomy was assigned, and abundance was calculated at all possible taxonomic levels. Diversity indices (Shannon and Chao1 indices), together with β-diversity matrices [i.e., weighted UniFrac and principal coordinate analyses (PCoA)] and rank abundance analysis, was computed using DADA2 (Callahan et al., 2016), phyloseq (McMurdie and Holmes, 2013), vegan (Oksanen et al., 2008), ape (Paradis et al., 2004), and phangorn (Schliep, 2011) R packages. Tidyverse, readxl, and Biostrings libraries of the R software package (http://www.r-project.org/) were also used throughout the pipeline. Sequences are available through PRJNA715367 and PRJNA715589 bio-projects numbers at National Center for Biotechnology Information (NCBI).

### *In vitro* Digestions

An *in vitro* digestion assay was performed on selected baked pieces of bread (eblfb1, eblfb2, and ebindb) as previously described by Mills et al. (2008), in order to mimic human digestive phases: mouth, stomach, and intestine. Briefly, 60 g of each bread were mixed with 150 mL of sterile distilled water and manually homogenized for 5 min. Afterward, 20 mg of α-amylase (A1031, Sigma–Aldrich) was mixed with 6.25 mL of CaCl_2_ 1 mM (pH 7.0) and subsequently added to the bread mixture and incubated at 37°C for 30 min under shaking at 150 revolutions/min (rpm). After incubation, pH was adjusted to 2.0 by adding HCl 1 M. Afterward, 25 mL pepsin solution (2.7 g of pepsin in 0.1 M HCl, PE0120, Pharmpur®) were added and incubated for 2 h or more at 37°C under the same shaking conditions. Finally, a bile and pancreatin solution (3.5 g bile (B8756, Millipore) and 560 mg pancreatin (P3292, Sigma–Aldrich) in 125 mL of NaHCO_3_ 30.5 M, pH 7.0) were added to each bread digested mixture. Once fully bread digestion was achieved, bread solutions were dialyzed through 100- to 200-Da cutoff membranes (Biotech CE dialysis, Spectrum Chemical Mfg. Corp.) overnight to remove monosaccharides. Finally, samples were centrifuged 4,500 *g* at 4°C for 10 min and supernatant eliminated. Pellets were stored at −20°C until further analyses.

Chemical parameters of bread predigestion and postdigestion were analyzed at external facilities (Laboratorio LINAS SL; Maçanet de la Selva, Spain).

### IBD Patient Recruitment

Fecal samples from a collection of IBD-diagnosed patients, specifically three samples from UC patients and three from CD patients, were selected from routine subject screening by clinicians of the Hospital Universitari de Girona Dr. Josep Trueta according to clinical criteria. Accordingly, patients were in an active state of the disease as defined by fecal calprotectin values (≥250 ng g^−1^). Fresh fecal samples were collected by participants in sterile feces' containers and delivered by participants to hospital facilities at room temperature <4 h after deposition. Samples were immediately analyzed [i.e., *in vitro* digestions and incubations, quantitative PCR (qPCR)] at GoodGut S.L. facilities (Girona, Spain) over the same day.

### Stool Samples *in vitro* Incubations

*In vitro* incubations of fecal homogenates with different bread conditions were performed to emulate the *in vivo* bacterial metabolism within the colon. Different amounts of substrates and fermentation buffer (FB; 0.1 M KH_2_PO_4_, 0.05 mM NaOH; pH 7.0) (Waldecker et al., 2008) were prepared to a final volume of 10 mL and dispensed into 20 mL borosilicate glass screw-cap tubes (from now on fermentation tubes). Before bread and fecal sample inoculation of tubes, fermentation buffer was degassed for 10 min at 100°C. Each experimental condition was run in triplicate. Incubation conditions were as follows: 20 mL of fermentation buffer (negative control); 10 mL of fermentation buffer (negative substrate control); 10 mL of fermentation buffer mixed with 1 g of Elias–Boulanger industrial bread (ebindb 1 g), 2 g of Elias–Boulanger industrial bread (ebindb 2 g), 1 g of Elias–Boulanger long-fermentation bread 1 (eblfb1 1 g), 2 g of Elias–Boulanger long-fermentation bread 1 (eblfb1 2 g), 1 g of Elias–Boulanger long-fermentation bread 2 (eblfb2 1 g), 2 g of Elias–Boulanger long-fermentation bread 2 (eblfb2 2 g), and 100 mg of pectin (positive control).

Fresh stool samples were weighted and mixed with FB to a concentration of 20% and manually homogenized in a sterile plastic bag for 2 min. Ten milliliters of the homogenized samples was added to the fermentation tubes. They were tightly sealed and incubated for 72 h at 37°C under 150 rpm shaking in anaerobic conditions. Negative incubation control was performed by mixing 10 mL of the homogenized samples with 10 mL of FB and directly stored at −20°C. Also, 100 mg of pectin were mixed with 10 mL of FB as a positive digestion control. All incubations were performed in anaerobic conditions. Aliquots were taken for subsequent qPCR determination of selected microbial species (see below).

### Quantitative PCR

Total genomic DNA was extracted from *in vitro* incubated bread samples using the NucleoSpin Soil Kit (Macherey-Nagel GMbH& Co.) following manufacturer instructions and eluted in 100 μL of elution buffer. Total genomic DNA was quantified using a Nanodrop ND-2000 UV-Vis spectrophotometer (Nanodrop, DE) and Qubit® (ThermoFisher Scientific®) measurements.

To quantify the abundances of representative bacterial representatives present in the gut microbiome, quantitative PCR was carried out targeting eubacteria (EUB) as the total bacterial load, *E. coli* (ECO), *F. prausnitzii* (FPRA), *F. prausnitzii* phylogroup I (PGHI) and phylogroup II (PGHII), *Akkermansia muciniphila* (AKK), *Roseburia* species (ROS), *Ruminococcus* species (RUM), *Clostridium* cluster XIV (XIV), *Lactobacillus* (LAC), Firmicutes (FIR), Bacteroidetes (BAC) groups, and species with best match BLAST related to *Subdoligranulum variabile* (B46).

Quantification of specific bacterial markers associated with IBD (i.e., FRA, ECO, PHGI, and PHGII) was performed by preparing a single reaction for each biomarker using Probe Master Mix (Promega, Madison, WI, USA) and TaqMan chemistry. Each reaction consisted of 10 μL containing 1× GoTaq qPCR Master Mix, between 300 and 900 nM of each primer, between 150 and 300 nM of probe (according to primer and probe combination, Table 1), and up to 20 ng of genomic DNA template. For all other bacterial markers targeted, specific reactions were prepared using SYBR Green Master Mix (Promega). Each reaction consisted of 10 μL containing 1× GoTaq qPCR Master Mix, between 150 and 300 nM of each primer (according to primer combination, Table 2), and up to 20 ng of genomic DNA template. Primers used in this study were purchased at Macrogen (Macrogen, Seoul, South Korea). All quantitative PCRs were run on an AriaMx Real-time PCR System (Agilent Technologies, Santa Clara, CA, USA). Thermal profiles were different and adjusted to each analyzed marker (Tables 1, 2). A melting curve step was added to the end of each thermal profile to verify the presence of the expected amplicon size and control primer dimer formation in SYBR Green reactions. Data were collected and analyzed with the Aria Software version 1.3 (Agilent Technologies). All samples were amplified in duplicates, which were considered valid when the difference between threshold cycles (Ct) was <0.6. No-template control (i.e., no DNA loaded) reactions were included in each qPCR run.

**Table 1:** 16S rRNA-targeted primers and probes sequences, concentrations, and qPCR conditions used for IBD-associated bacterial markers in this work for TaqMan chemistry.

Target^*^	**Primers and probes ^a^**				**qPCR conditions**			
					**Denaturing**		**Annealing (40 cycles)**	
	**Primers**	**Concentration (nM)**	**Sequence (5^′^-3^′^)**	**Reference**	**Time (min:s)**	***T*** **^a^ (^°^C)**	**Time (min:s)**	***T*** **^a^ (^°^C)**
FPRA	Fpra_F Fpra_R	250	TGTAAACTCCTGTTGTTGAGGAAGATAA GCGCTCCCTTTACACCCA	(Lopez-siles et al., 2014)	2:00 10:00	50 95	0:15 1:00	95 60
	**Fpra_PR**	**300**	**FAM-CAAGGAAGTGACGGCTAACTACGTGCCAG-TAMRA**					
ECO	Eco_F	300	CATGCCGCGTGTATGAAGAA	(Round and Mazmanian, 2009)	2:00 10:00	50 95	0:15 1:00	95 60
	Eco_R		CGGGTAACGTCAATGAGCAAA					
	**Eco_PR**	**100**	**FAM-TATTAACTTTACTCCCTTCCTCCCCGCTGAA-TAMRA**					
PHGI PHGII	PHG_F PHG_R	300	CTCAAAGAGGGGGACAACAGTT GCCATCTCAAAGCGGATTG	(Lopez-siles et al., 2016)	2:00 10:00	50 95	0:15 1:00	95 64
	**PHGI_PR**	**900**	**FAM-TAAGCCCACGACCCGGCATCG-BHQ1**					
	**PHGII_PR**		**JOE-TAAGCCCACRGCTCGGCATC-BHQ1**					

* *EUB, Eubacteria; FPRA, F. prausnitzii; ECO, E. coli; PHGI, F. prausnitzii phylogroup I; PHGII, F. prausnitzii phylogroup II; NA, non-applicable.*

a *Probe sequences are in bold font. FAM™, 6-carboxyfluorescin; JOE, 4′,5′-dichloro-2′,7′-dimethoxy-5(6)-carboxyfluorescein; TAMRA™, tetramethylrhodamine; BHQ1, Black Hole Quencher1*.

**Table 2:** 16S rRNA-targeted primers sequences, concentrations, and qPCR conditions used in this work for SYBR green chemistry.

Target^*^	**Primers**	**Concentration** **(nM)**	**Sequence (5^′^-3^′^)**	**Reference**	**qPCR conditions**					
					**Denaturing**		**Annealing (40 cycles)**		**Melting curve**	
					**Time (min:s)**	***T*** **^a^ (^°^C)**	**Time (min:s)**	***T*** **^a^ (^°^C)**	**Time (min:s)**	***T*** **^a^ (^°^C)**
EUB	Eub_F Eub_R	200	ACTCCTACGGGAGGCAGCAGT GTATTACCGCGGCTGCTGGCAC	(Matsuda et al., 2011)^¶^	10:00	95	0:15 1:00	95 54	1:00 0.30	95 55
									0:30	95
AKK	AKK_F AKK_R	250	CAGCACGTGAAGGTGGGGAC CCTTGCGGTTGGCTTCAGAT	(Sekirov et al., 2010)	10:00	95	0:15 1:00	95 60	1:00 0.30	95 55
									0:30	95
ROS	ROS_F ROS_R	150	TACTGCATTGGAAACTGTCG CGGCACCGAAGAGCAAT	(Larsen et al., 2010)	10:00	95	0:15 1:00	95 60	1:00 0.30	95 55
									0:30	95
RUM	RUM_F RUM_R	250	GGCGGCYTRCTGGGCTTT CCAGGTGGATWACTTATTGTGTTAA	(Round and Mazmanian, 2009)	10:00	95	0:15 1:00	95 60	1:00 0.30	95 55
									0:30	95
LAC	VLAC_F VLAC_R	200	AGCAGTAGGGAATCTTCCA CGCCACTGGTGTTCYTCCATATA	(Payne et al., 2012)	10:00	95	0:15 1:00	95 60	1:00 0.30	95 55
									0:30	95
FIR	FIR_F FIR_R	150	TGAAACTYAAAGGAATTGACG ACCATGCACCACCTGTC	(Mühling et al., 2008)	10:00	95	0:15 1:00	95 60	1:00 0.30	95 55
									0:30	95
BAC	BAC_F BAC_R	300	CRAACAGGATTAGATACCCT GGTAAGGTTCCTCGCGTAT	(Lozupone et al., 2012)	10:00	95	0:15 1:00	95 60	1:00 0.30	95 55
									0:30	95
XIV	VXIV_F VXIV_R	300	CGGTACCTGACTAAGAAGC AGTTTYATTCTTGCGAACG	(Ramirez-Farias et al., 2009)	10:00	95	0:15 1:00	95 60	1:00 0.30	95 55
									0:30	95
B46	B46_F B46_R	300	TCCACGTAAGTCACAAGCG CGCCTACCTGTGCACTACTC	(Malagón et al., 2019)	10:00	95	0:15 0:45	95 62	1:00 0.30	95 55
									0:30	95

* *AKK, Akkermansia muciniphila; ROS, Roseburia species; RUM, Ruminococcus species; LAC, Lactobacillus; FIR, Firmicutes; BAC, Bacteroidetes; XIV, Clostridial cluster XIV; B46, S. variabile.*

¶ *Modified from*.

### SCFA Analyses

After separating aliquots for qPCR assay, *in vitro* incubation tubes were centrifuged at 4,500 *g*, 4°C for 30 min. Supernatants were recovered in new 15 mL Falcon tubes and further centrifuged at 4,500 *g* at 4°C for 15 min. Afterward, supernatants were sterilized by syringe filtration using 0.22-μm-pore-size filters. Acetic, propionic, butyric, isobutyric, valeric, isovaleric, and hexanoic acids were analyzed using a gas chromatograph (Agilent 7890A GC system; Agilent Technologies) equipped with a fuse-silica capillary column (DB-FFAP, 30 m × 0.32 mm × 0.5 μm) and a flame ionization detector. All the analyses were performed at the Laboratory of Chemical and Environmental Engineering of the University of Girona (LEQUIA UdG; Girona, Spain).

### Statistical Analyses

All statistical analyses were performed using SPSS 23.0 statistical package (IBM, Armonk, NY, USA). Data normality was assessed through the Kolmogorov–Smirnov test. Pairwise comparisons of subcategories of these variables were analyzed using a Mann–Whitney non-parametric test. Significance levels were established for *p* ≤ 0.05. Statistical analyses of qPCR and SCFA data were performed among samples with the same conditions (i.e., UC or CD).

## Results

### Microbial Diversity

**Figure 1:**
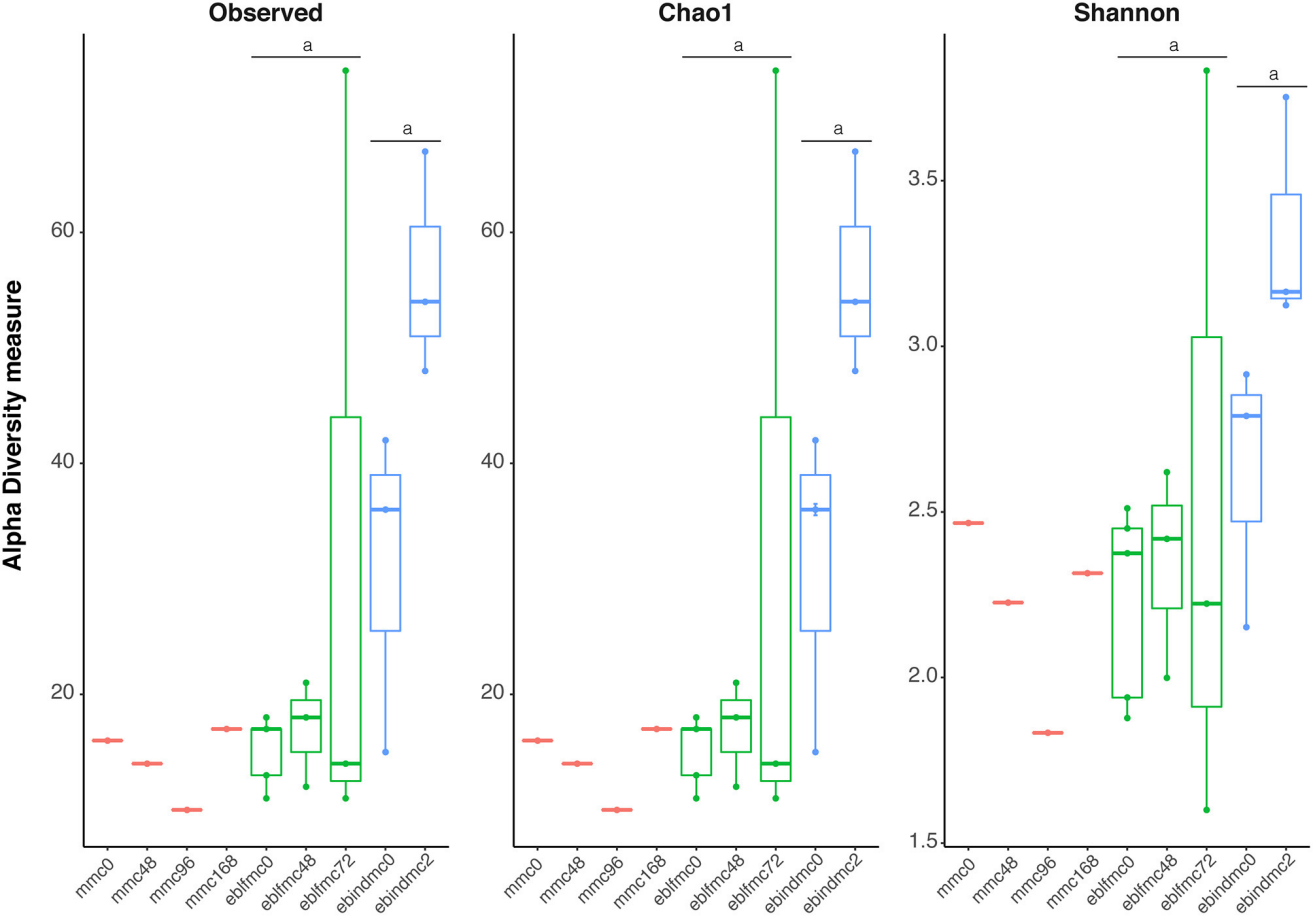
Bacterial richness and diversity indices (16S rRNA gene) obtained from the massive sequencing analysis of the different types of bread doughs (MMC, sourdough; eblfmc, Elias–Boulanger long-fermentation raw dough; ebindmc, Elias–Boulanger industrial raw dough) at different times of fermentation. Statistical comparisons between eblfmc and ebindmc groups are indicated by superscript letters: same letters indicate statistically significant differences (*p* < 0.05).

Non-parametric species richness estimates (Chao1) and diversity indices (Shannon) for bacteria (Figure 1) showed slight variations among different times of fermentation in MMC samples, with decreasing in richness and diversity indices until 96 h of fermentation (mmc96) but increasing at 168 h (mmc168) to almost the same levels as at 0 h of fermentation (mmc0). Unfortunately, only one sample per fermentation time of MMC was obtained, and no statistical support was achieved. Nonetheless, ebindmc samples showed significantly higher levels of species richness (*p* < 0.027) and diversity (*p* < 0.037) compared to eblfmc samples. However, no significant differences were observed when comparing bread doughs at different fermentation times [i.e., 0 h. (ebindmc0 vs. eblfmc0) or between different fermentation stages (ebindmc2 vs. eblfmc48 and ebindmc2 vs. eblfmc72)].

**Figure 2:**
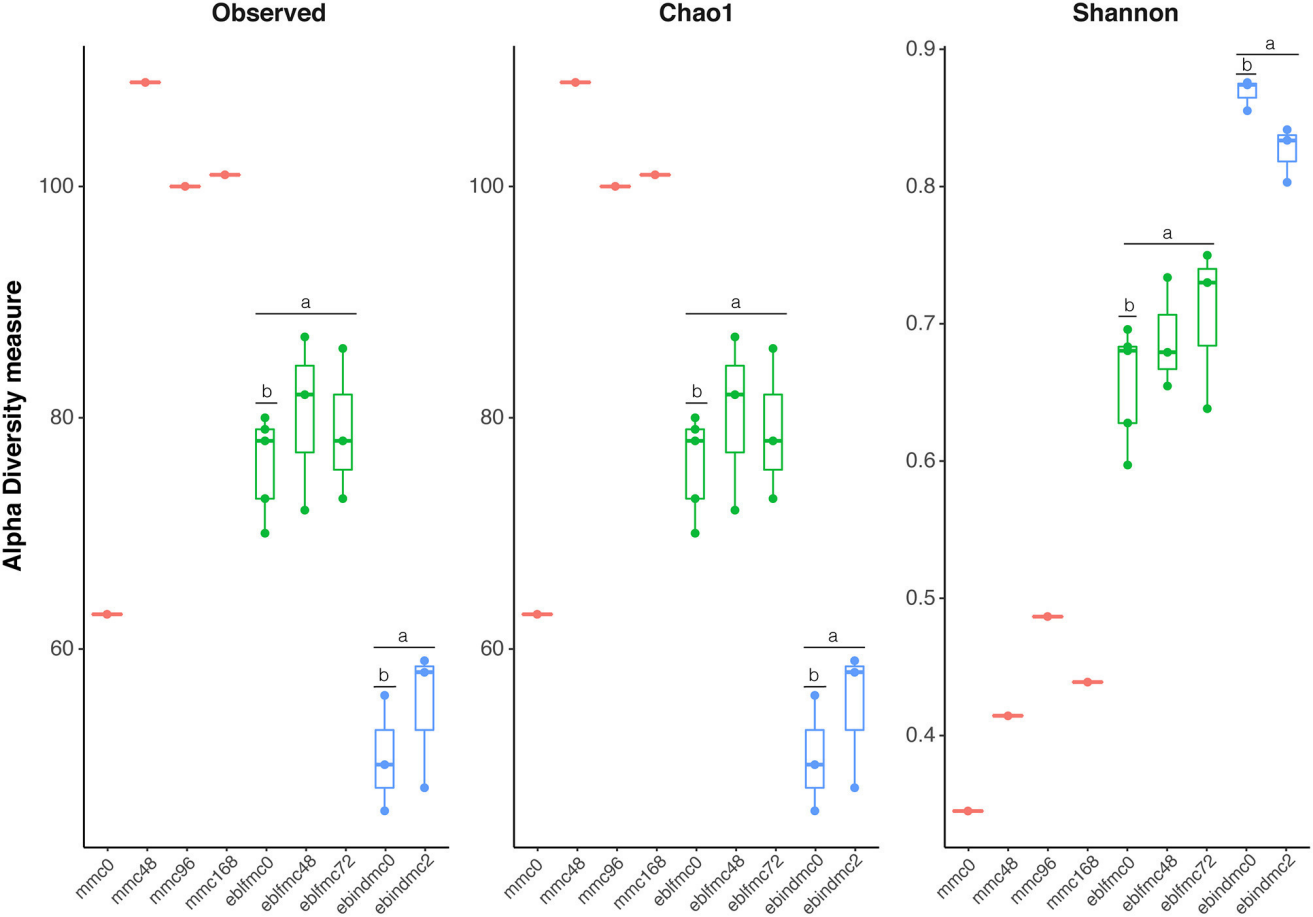
Yeasts' richness and diversity indices (18S rRNA gene) obtained from the massive sequencing analysis of the different types of bread doughs (MMC, sourdough; eblfmc, Elias–Boulanger long-fermentation raw dough; ebindmc, Elias–Boulanger industrial raw dough) at different times of fermentation. Statistical comparisons between eblfmc and ebindmc groups are indicated by superscript letters: same letters indicate statistically significant differences (*p* < 0.05).

Contrarily, results for yeast (Figure 2) showed that MMC samples after 48 h of fermentation had the greatest species richness (no statistical support due to the lack of replicates), and followed by eblfmc samples, which in turn showed significantly (*p* < 0.001) higher species richness levels than ebindmc samples. However, in terms of diversity, the results were the opposite. Ebindmc samples showed significantly (*p* < 0.001) higher values than eblfmc samples. MMC diversity values increased with longer fermentation times, although with much lower values than in bread dough samples. Furthermore, significant differences between initial fermentation stages in both flours (i.e., ebindmc0 and eblfmc0) were found for both richness (*p* < 0.036) and diversity (*p* < 0.036) indices, but not between longer fermentation periods (i.e., eblfmc48 or eblmc72 with respect to ebindmc2). Such diversity richness differences between samples were not caused by different amount of DNA present in analyzed samples, ranging from 7.4 × 10^4^ to 9.7 × 10^6^ copies of eubacterial 16S rRNA gene per gram of bread dough (Supplementary Figure 1).

Rank abundance analyses of the top 20 retrieved ASVs for bacteria (Supplementary Figure 2) showed that ASV13 was the only one found in all sample groups and shared a 100% pairwise sequence identity with *Curtobacterium flaccumfaciens* strain YZYP506 (MN931343). The two most detected ASVs [ASV6 (99.75% similar to *Phyllobacterium myrsinacearum* strain J5R2LARS (MT378449)] and ASV11 [100% similar to *Rhodococcus qingshengii* strain 367-6 (MT632489); 11.21% and 7.15% of total sequences, respectively), were mainly present in ebindmc samples. However, many of the top 20 ASVs (i.e., ASV36, ASV38, ASV35, ASV46, and ASV58) had similarities between 98.93 and 99.64% to *L. sanfranciscensis* strain LS451 (CP045563), accounting for up to 19.70% of the total sequences. These ASVs were mainly found in elbfmc and MMC samples. ASV29 [100% similar to *Sphingomonas olei* strain 2 (MT415749)] and ASV34 [96.03% similar to *Paenibacillus nuruki* strain TI45-13ar (MH707258)] were found in almost all types of bread doughs at different times of fermentation. Unlike bacterial results, two yeast ASVs accounted for up to 95.96% (ASV1 and ASV2; 80.20 and 15.76%, respectively), of the total retrieved 18S rRNA gene sequences. AVS1 [100% similar to *T. dicoccoides* (XR_005169686) and *Aegilops tauschii* subsp. *strangulata* (XR_005764925)] was found at similar abundances in all groups, whereas ASV2 [100% similar to *S. cerevisiae* strain ySR128 (CP036478)] was found only in bread dough groups (i.e., eblfmc and ebindmc) at similar abundances (Supplementary Figure 3).

**Figure 3:**
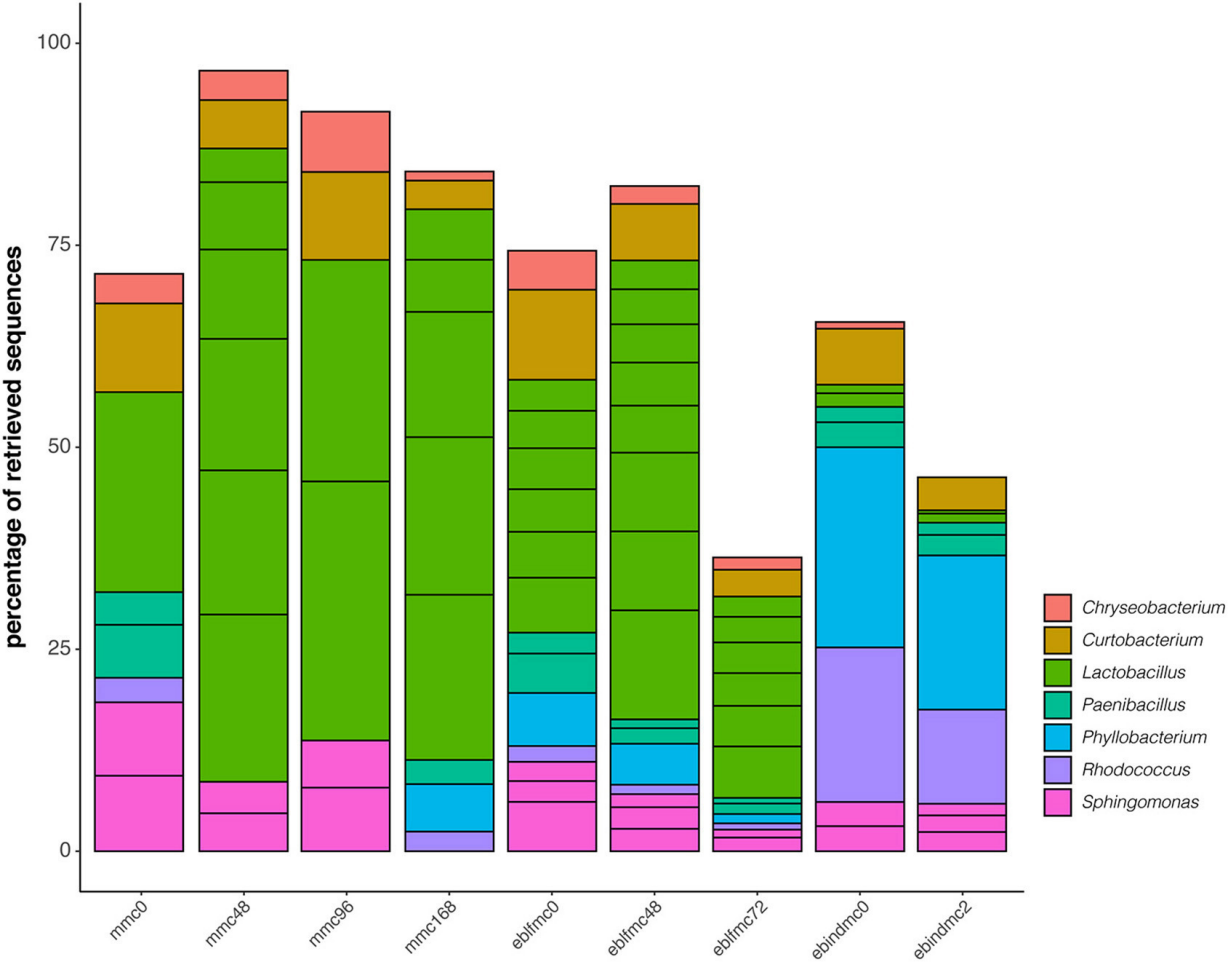
Phylogenetic diversity of the first 20 most abundant ASVs for bacteria (16S rRNA gene) recovered from the massive sequencing analysis of the different types of bread doughs (MMC, sourdough; eblfmc, Elias–Boulanger long-fermentation raw dough; ebindmc, Elias–Boulanger industrial raw dough) at different times of fermentation.

The top 20 retrieved ASVs for bacteria were classified into seven different genera (Figure 3). *Lactobacillus, Curtobacterium, Paenibacillus*, and *Sphingomonas* were the main genera found at time 0 of the sourdough fermentation (mmc0); *Chryseobacterium* and *Rhodoccocus* were also identified at lower frequencies. Soon after the first fermentation stage, the bacterial profile markedly changed and became mainly dominated by *Lactobacillus* followed by a decrease of *Paenibacillus* and *Rhodococcus*, which were neither identified at 48 or 96 h of sourdough fermentation. In samples taken after 168 h, *Paenibacillus, Rhodoccocus*, and *Phyllobacterium* were identified instead of *Sphingomonas*. Once sourdough was mixed with whole-grain wheat flour to produce elbfmc, *Lactobacillus* predominance was less critical, and *Sphingomonas* was again detected. After 48 and 72 h of eblfmc fermentation, a similar pattern occurred, where *Lactobacillus* strongly predominated, and almost all other genera abundances decreased.

**Figure 4:**
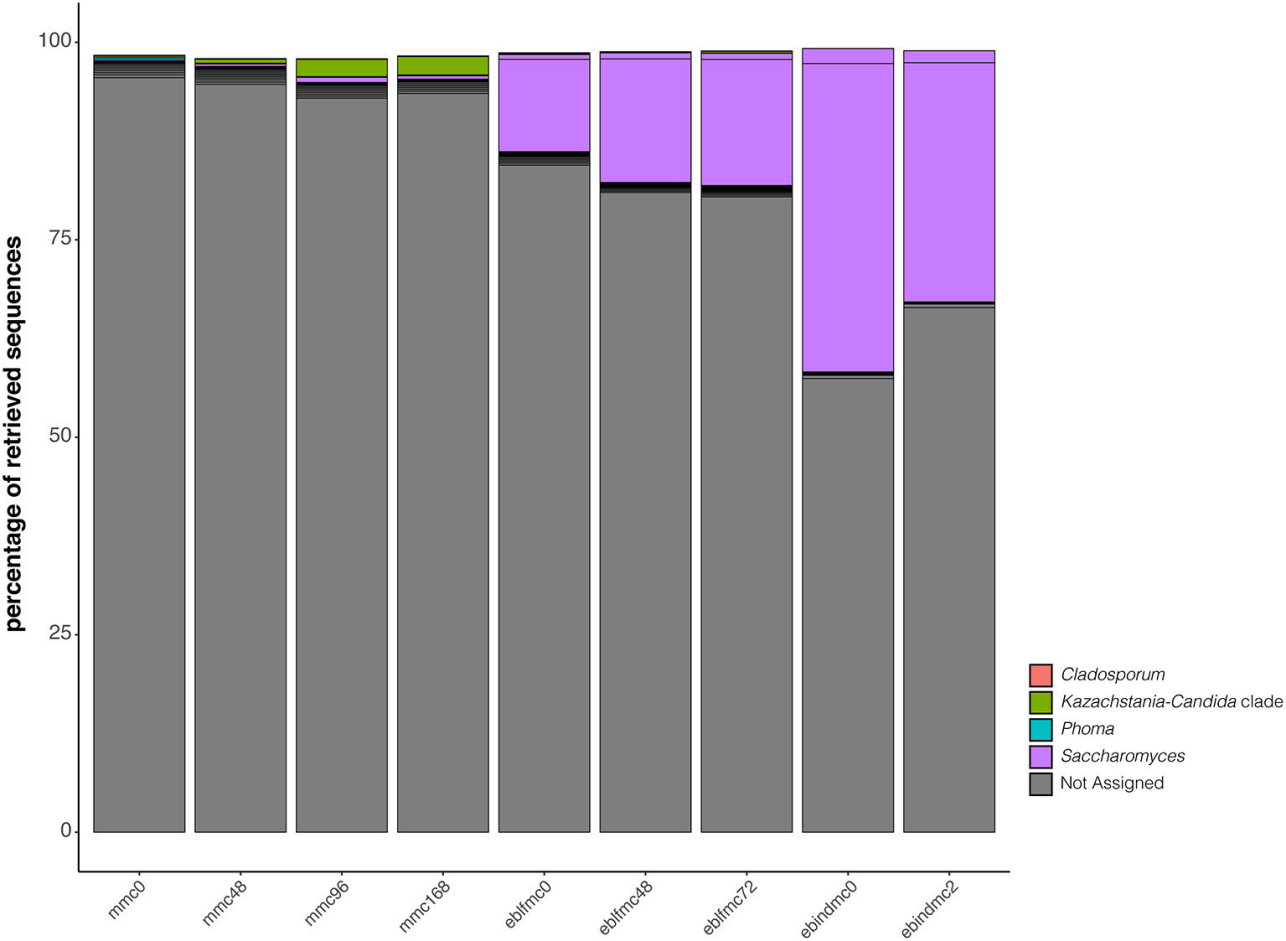
Phylogenetic diversity of the first 20 most abundant ASVs for yeasts (18S rRNA gene) recovered from the massive sequencing analysis of the different types of bread doughs (MMC, sourdough; eblfmc, Elias–Boulanger long-fermentation raw dough; ebindmc, Elias–Boulanger industrial raw dough) at different times of fermentation.

Conversely, the two most representative genera in ebindmc groups were *Phyllobacterium* and *Rhodococcus*. Even at low relative abundance values, *Lactobacillus, Chryseobacterium, Curtobacterium, Paenibacillus*, and *Sphingomonas* related ASVs were also found. Besides, a similar bacterial community was observed for ebindmc samples at both analyzed fermentation times. In yeasts (Figure 4), a non-classified bacterium at genus level dominated all dough groups. In turn, *Saccharomyces* was found at low incidence in the initial sourdough (MMC) but lightly increased as the fermentation time progressed. Interestingly, results showed a higher incidence of *Saccharomyces* in dough groups than in sourdough groups, being even more noticeable in ebindmc groups. *Phoma, Kazaschtania-Candida_clade*, and *Cladosporium* were also identified, although at very low values.

PCoAs based on the weighted UniFrac distance matrixes are shown in Supplementary Figures 4, 5 for both analyzed targets (i.e., 16S rRNA and 18S rRNA gene). The two principal coordinates for bacteria (Supplementary Figure 4) explained 89.1% of the total variability of the data. The distribution of dough samples on the plot was mainly related to the type of dough. Samples from the same dough type clustered together through axis 1, representing 80.3% of total variability. As for yeasts (Supplementary Figure 5), the first axis explained 99.8% of the data variability. Groups clustered in three areas that correlated with the type of dough rather than the fermentation time.

### Breads Nutritional Parameters

Chemical and nutritional parameters of the tested baked bread types were analyzed and shown in Table 3. Although slight differences were appreciated in all analyzed parameters, and even statistically significant differences in moisture, ash, calories, calcium, and potassium values among types of bread (*p* ≥ 0.05 in Kruskal–Wallis non-parametric test) were found, lack of statistical power (*n* = 3) prevented detection of significant differences when multiple comparisons were performed.

**Table 3:** Chemical properties of the three different tested breads.

**Components**	**Ebindb (*n =* 3)**	**Eblfb1 (*n =* 3)**	**Eblfb2 (*n =* 3)**
Proteins^a^	10.12 ± 0.17	8.12 ± 0.20	9.09 ± 1.15
Dietary fiber^a^	2.60 ± 0.17	3.13 ± 0.32	3.27 ± 0.47
Moisture^a^	33.60 ± 0.50	36.00 ± 0.78	33.93 ± 1.68
Ash^a^	1.59 ± 0.03	2.32 ± 0.61	2.14 ± 0.16
Carbohydrates^a^	51.47 ± 0.06	49.43 ± 1.17	50.43 ± 2.04
Calories^b^	257.67 ± 1.53	245.33 ± 5.51	254.33 ± 4.93
Sodium^a^	0.50 ± 0.01	0.74 ± 0.24	0.64 ± 0.05
Potassium^c^	1.27 ± 0.06	1.57 ± 0.06	1.63 ± 0.12
Magnesium^c^	0.29 ± 0.01	0.40 ± 0.01	0.41 ± 0.05
Calcium^c^	0.20 ± 0.00	0.27 ± 0.00	0.28 ± 0.03

a *Values are grams per 100 g of sample.*

b *Values are micrograms per liter of sample.*

c *Values are grams per kilogram of sample.*

Some parameters (i.e., protein, dietary fiber, starch, and SCFAs) of bread after being *in vitro* digested were also analyzed and represented in Supplementary Table 1.

### Bacterial Markers Abundance on Feces

The relative abundance of bacterial markers of UC patients showed that EUB, BAC, and XIV biomarkers abundances increased in all conditions compared to substrate control (Supplementary Table 3). FIR abundance was also increased in all conditions, except for incubations with 1 g of eblfb1. On the contrary, FPRA was favored by longer fermentation times, and abundance of FPRA phylogroups was mainly increased after incubations with low doses regardless of the type of bread. Other bacteria such as RUM and AKK were more abundant in treatments with bread than with pectin, regardless of the quantity of incubated bread. ROS also reached higher abundances when incubated with 2 g, whereas B46 displayed higher numbers at 1 g of incubated bread. Interestingly, ECO presented higher accounts when incubated with eblfb2, ebindb, and pectin.

Concerning CD patients, EUB, AKK, and RUM marker abundances increased in all conditions compared to substrate control (Supplementary Table 2). The same results were found in BAC marker abundance, although the highest amounts were achieved with 2 g of ebindb. Moreover, ECO, ROS, FIR, and XIV abundances also increased with 2 g of bread regardless of bread type. No differences among different treatments or doses were found for FPRA, PHGI, PHGII, or B46.

### SCFA Concentration on Feces

As shown in Supplementary Table 4, most SCFA concentrations increased in all different conditions tested. However, neither significant differences in isobutyric acid nor any increase in concentrations of isovaleric acid was found. Acetic, propionic, and butyric acids were increased up to 3-fold in bread treatments compared to substrate control. Concentrations of these compounds were higher at 2-g incubations as compared to 1 g ones. Valeric acid also increased in bread treatments as compared to substrate controls, but no relevant differences were found between bread quantities. Hexanoic acid was increased in 2 g dose of eblfb2 and ebindb when compared to substrate control. Finally, there were no differences among bread treatments at the same bread quantity.

In the case of patients suffering from CD (Supplementary Table 5), all SCFAs analyzed were higher after incubations with bread than in control cases. Acetic, propionic, and butyric acid concentrations were more than 2-fold increased after incubating with different kinds of bread. Moreover, the highest increases were found with 2-g dose. Treatments with 2 g of all three types of bread showed almost up to 4-fold higher SCFA concentration than substrate control. As observed with bacterial counts, no relevant differences on SCFAs were found among different tested types of bread when the same dose was used, which agrees with the results of the biological markers.

## Discussion

Bread-making process is recognized to make a big impact on our nutrition and health, depending on various factors such as dough composition and fermentation. The interest in investigating bread proprieties and qualities is growing worldwide because of its possible effects on a well-balanced diet and its capacity as a therapeutic dietary intervention (Korem et al., 2017; Abbondio et al., 2019).

Recently, several studies have considered the influence of different factors on the microbial composition of sourdough and consequently on final bread properties. For instance, the wheat species (Minervini et al., 2012b), the cereals used (Vogelmann et al., 2009), the temperature and back-slopping time (Vrancken et al., 2011), the location of the propagation (artisan bakery or laboratory) (Minervini et al., 2012a), and technological factors (Vogelmann and Hertel, 2011) have been found to alter dough microbial composition. Although sourdoughs' microbial diversity has been largely studied (Scheirlinck et al., 2007; Vogelmann et al., 2009; Valmorri et al., 2010; Vogelmann and Hertel, 2011; Minervini et al., 2012b; Huys et al., 2013; Boreczek et al., 2020; Comasio et al., 2020), few studies have investigated microbial composition shifts throughout sourdough fermentation in back-slopping stages or among distinct bread doughs in terms of chemical composition and fermentation times. From our knowledge, only few studies analyzed the bacterial community dynamics along the sourdough fermentation process by high-throughput sequencing (Ercolini et al., 2013; Boreczek et al., 2020; Comasio et al., 2020).

Alpha diversity results hinted a higher bacterial richness and diversity in Elias–Boulanger industrial raw doughs and higher yeast diversity compared to the ones containing sourdough (Figures 1, 2). On the contrary, the highest levels of yeasts in terms of diversity and richness seemed to be found in sourdough fermented for more than 48 h, whereas the lowest levels corresponded to the Elias–Boulanger industrial raw doughs. The microbial community involved in the sourdough fermentation process usually includes *Lactobacillus*-related species and fungi, particularly yeasts (De Vuyst et al., 2014; Vuyst et al., 2016; Carbonetto et al., 2018). Therefore, lower bacteria and yeast diversity in samples containing sourdough was already expected. It is worth mentioning that most of the retrieved sequences belonged to plants (i.e., *T. dicoccoides* and *A. tauschii* subsp. *strangulata*), thus masking yeast recovery. Further, comparisons between bread doughs at time 0 provided differences in yeasts richness and diversity but not in the case of bacteria, most likely due to lack of size of the sample. Probably, the lack of statistical power also prevented us from observing differences in richness and diversity between bread doughs at different fermentation times.

Initial sourdough was evenly inhabited by Firmicutes (i.e., *Lactobacillus* and *Paenibacillus*), Actinobacteria (i.e., *Curtobacterium*, and *Rhodococcus*), Bacteroidetes (i.e., *Chryseobacterium*), and Proteobacteria (i.e., *Sphingomonas*). Actinobacteria and Bacteroidetes have been reported to dominate the wheat root endosphere (Ding et al., 2013), and Proteobacteria are found in wastewater, forage feed, and soils (Andra, 2012). After 48 h of fermentation, *Lactobacillus* predominated above all other genera, although C*hryseobacterium, Curtobacterium*, and *Sphingomonas* were still identified. This predominance remained through the different stages of sourdough fermentation. Among other genera, *Lactobacillus* and *Paenibacillus* have been described as some of the Firmicutes inhabiting wheat flour (Ercolini et al., 2013). De Vuyst et al. (2014) evidenced that *Paenibacillus* could not colonize or survive in bakery equipment, and thus, they are not always identified in sourdoughs. On the other hand, *Lactobacillus* was the genus with the highest adaptability to bakery equipment and sourdough.

The most dominant 18S ASV in all groups was only classified at class level (i.e., *Embryophyta*); thus, we were unable to reach species level. Sequence similarity of most abundant retrieved 18S ASV (i.e., ASV1) was assigned to *T. dicoccoides* and *A. tauschii* subsp. *strangulata*, both commonly used as wheat for flour and bread production. It is worth mentioning that common wheat hexaploid species [*T. aestivum* ssp. *spelta* (AABBDD) and *T. aestivum* ssp. *aestivum* (AABBDD)] contain genome fragments of ancient *Triticum* family representatives [*T. dicoccoides* (AABB), *A. tauschii* (DD)] (Kucek et al., 2015). In the present study, sourdough was elaborated by mixing water and whole-grain wheat (*T. dicoccoides*) flour, whereas the Elias–Boulanger long-fermentation raw dough was prepared mixing sourdough product and whole-grain wheat (*T. aestivum*) flour. However, *S. cerevisiae* was also identified in all groups. This yeast group was found at very low values in the initial sourdough, but as back-slopping time evolved, *S. cerevisiae* abundance increased. By definition (Ercolini et al., 2013), the continuous sourdough refreshments aim at selecting LAB and yeasts. Among LAB, *Pediococcus, Leuconostoc, Weissella*, and especially *Lactobacillus* genera, have been identified frequently (Huys et al., 2013). Among yeasts, the species *S. cerevisiae* and *Candida humilis* are the most common taxa found (Minervini et al., 2012a). Going deeper into LAB species, unlike previous studies (Vera et al., 2012), *L. sanfranciscensis* was found to dominate the sourdough ecosystem unquestionably, yet it was the only LAB species identified. A previous report (Vogel et al., 2011) on *L. sanfranciscensis* genome evidenced its capacity to develop in traditional sourdough ecosystems readily and outcompete other bacterial groups present (Vogel et al., 2011).

A similar pattern occurred for Elias–Boulanger long-fermentation raw dough, made up with sourdough. In this case, the same bacteria were evenly inhabiting the mixture at start-up, but after 48 and 72 h of fermentation, the bacterial structure was dominated by *L. sanfranciscensis*. However, noticeable differences in bacterial predominance were detected in Elias–Boulanger industrial raw dough. *Phyllobacterium* (within Proteobacteria phylum) and *Rhodococcus* (within Actinobacteria phylum) were the main predominant genera, both before and after 2-h fermentation. *Phyllobacterium* has been identified in several different environments, but their presence was primarily described in plant tissues, including leaf or root nodules (Mantelin et al., 2006). *Rhodococcus* has been reported in wheat rhizospheres (Kavamura et al., 2019). As is common practice in bread production strategies, *Saccharomyces* was more abundant in bread doughs than in sourdough samples as dried baking yeasts (*S. cerevisiae* strain) were added to prepare both bread doughs.

Weighted UniFrac distance matrices differentiated groups according to the type of dough and sourdough for both analyzed genes (i.e., 16S rRNA and 18S rRNA), regardless of fermentation time. These results suggested that the primary variable influencing the bacterial composition of raw bread doughs is using sourdough as a starter fermenter. In contrast, the time of fermentation in raw bread doughs did not seem to have any apparent influence or impact on bacterial or yeast community composition.

During sourdough fermentation, several factors such as acidification, proteolysis, and activation of some enzymes and the synthesis of microbial metabolites cause changes in the dough and positively influence its sensory, nutritional, and functional features (De Vuyst et al., 2009; Gobbetti et al., 2014). LAB enzymatic activities in sourdough may contribute to an increase in the quantity of soluble fibers in food products. Fiber solubilization is correlated with flour endogenous and microbial enzymes, such as xylanases (Katina et al., 2006) that, acting on arabinoxylans, allow the solubilization of the insoluble fiber fraction (Montemurro and Coda, 2019). It is noteworthy that the most decisive fiber to condition the intestinal microbiota and its fermentation products is soluble fiber (Montemurro and Coda, 2019). Dietary fiber is a complex carbohydrate known to promote the production of SCFAs through fermentation by the resident anaerobic colonic microbiota in the gut (Macfarlane and Gibson, 1997). SCFAs, in turn, play an essential role in maintaining the health of colonic mucosa through regulation of the intestinal epithelial cell growth, as well as differentiation and stimulation of the immune system, among other biological processes. Besides, a lower glycemic index was measured in sourdough bread, which leads to a higher amount of slowly digestible and resistant starch that reaches the colon, where it is degraded by colonic bacteria to produce SCFAs. Bread is a universal food consumed all over the world that contains a considerable amount of dietary fiber. Also, the fermentation process has been seen to influence the final dietary fiber composition (Saa et al., 2017) profoundly.

Interestingly, the nutritional parameters of final products elaborated for this study showed that, although it was not statistically supported in postdigested bread, there were some differences in composition among bread types. The Elias–Boulanger industrial bread was the lowest in dietary fiber, both before (Table 3) and after being *in vitro* digested (Supplementary Table 1). In contrast, the Elias–Boulanger long-fermentation bread fermented for 72 h was the one with the highest amounts of dietary fiber (Table 3 and Supplementary Table 1). Bread doughs composition and fermentation might be the main culprits of these results.

Results were separately analyzed according to disease, UC or CD, as it is well-established that the microbiota found in these diseases differ from one another. Results for both UC and CD showed significant differences in the abundances of the analyzed bacterial markers and SCFA production. In both diseases, abundances of most bacteria (qPCR) were increased compared to negative controls, suggesting a modification of the gut microbiome according to treatment. It is worth mentioning that, without taking into account the total bacterial load (EUB quantifications), *E. coli* (a proinflammatory bacterium), and *Lactobacillus* (which has discrepancies in terms of abundance shifts in IBD patients), all other bacterial markers analyzed are commonly reduced in terms of abundance in IBD patients compared to healthy ones (Wang et al., 2014; Rivière et al., 2016; Nagao-Kitamoto and Kamada, 2017; Lopez-Siles et al., 2018). Therefore, obtained changes in the composition of the colonic environment may indicate a trend toward the partial recovery of the gut bacterial ecology, similar to that found in healthy subjects.

Above all SCFAs, acetic, butyric, and propionic acids are the three main luminal SCFAs produced in the human gut (60:20:20 mM/kg) (Martin-Gallausiaux et al., 2020). Propionic and acetic acids have been shown to possess anti-inflammatory properties and have been found in lower concentrations in UC stool samples (Machiels et al., 2014). Nonetheless, butyric acid is, by far, the most extensively studied SCFAs, and its anti-inflammatory capacity is well-documented both *in vitro* and *in vivo*. Notably, several clinical studies demonstrate beneficial effects of butyric acid in IBD (Scheppach et al., 1992; Steinhart et al., 1996; Di Sabatino et al., 2005). In the present study, all studied conditions for both UC and CD fecal samples produced an increase in abundance of all butyrate-producing species studied: *F. prausnitzii, Ruminococcus* species, *Roseburia* species, *Clostridium* cluster XIV, and *S. variabile*, which are commonly found at low abundances in unhealthy guts, such as in IBD (Rivière et al., 2016; Nagao-Kitamoto and Kamada, 2017; Lopez-Siles et al., 2018). However, less clear results regarding which type or dose of bread prompts a major increase of butyrate producers were achieved.

Nonetheless, according to bacterial abundances and metabolic properties, we want to highlight the increase of *Roseburia* species abundance, as it seems to be the main responsible for enhancing butyric, propionic, and acetic acid production. *Roseburia* species are known to ferment complex polysaccharides to butyrate as a terminal product in the colon (Tamanai-Shacoori et al., 2017). Although there is little evidence to suggest that these species regularly ferment to propionate, *Roseburia* appears to possess the genetic capacity to convert precursor compounds to propionate via a propanediol utilization (*pdu*) operon (Scott et al., 2006). In addition, *Roseburia* species uniformly has the necessary genes to produce acetate. Although it is not likely a major final fermentation product, a net consumption of acetate has been observed by Hillman et al. (2020), which is essential in the butyrate fermentation strategy. Some studies observed that cereal brans increase butyrate-producing Clostridia, especially *Roseburia* species (Leitch et al., 2007; De Paepe et al., 2018, 2020). Distinctly from our results, an *in vivo* study showed that intake of whole-grain diets compared to a more refined grain increased clostridial butyrate-producer *Roseburia* (Vanegas et al., 2017). Whole grains also increased fecal SCFA production in human studies (Vuholm et al., 2017). Our results are in concordance with other studies that described *Roseburia* species playing a major role in UC (Rajilić-Stojanović et al., 2013; Machiels et al., 2014). In these reports, a decrease in the butyrate-producing species *Roseburia* species and *F. prausnitzii* compared to healthy individuals was found. They also suggested the use of these species to restore and maintain the microbiota balance in UC patients with selective probiotics, prebiotics and symbiotic.

To conclude, this study provides new data on the overall microbial dynamics at different moments of fermentation in sourdough preparation, as well as on the microbial community of two kinds of bread doughs with different fermentation lengths before baking. Furthermore, this study is the first to describe and compare the impact of three bread products on the fecal microbiota community composition of IBD patients. Further investigations with larger cohorts and *in vivo* clinical studies are needed to verify this first overview of how sourdough combined with long fermentations in bread-making could be a potential tool to help restoring dysbiosis and gut health for IBD patients.

## Data Availability Statement

All relevant data within the present article and its supporting information are available upon request. All sequences generated in the present study are available and accessible through National Center for Biotechnology Information (NCBI) bioprojects number: PRJNA715367 and PRJNA715589.

## Ethics Statement

The studies involving human participants were reviewed and approved by Comitè d'Ètica d'Investigació Clínica Hospital Doctor Josep Trueta. The patients/participants provided their written informed consent to participate in this study.

## Author Contributions

AL: methodology, formal analysis, investigation, resources, writing—original draft, and visualization. ML: methodology, formal analysis, investigation, writing—review & editing, and visualization. LO: methodology, formal analysis, and investigation. AB: methodology and resources. NE-M, PB, EC, DB, LT, MSa, and MG: resources. SR-P: methodology, formal analysis, and investigation. MSe, MM, MC, and JA: investigation. MB: formal analysis. MS-P: conceptualization, methodology, and visualization. MT: resources and writing—review. SD-A: conceptualization, methodology, writing—review, and visualization. LJG-G: conceptualization, methodology, formal analysis, writing—review & editing, visualization, supervision, project administration, and funding acquisition. IE and XA: conceptualization, resources writing—review & editing, visualization, supervision, project administration, and funding acquisition. All authors contributed to the article and approved the submitted version.

## Funding

AL benefits from a grant included within the Ministry of Economy, Industry and Competitiveness (MINECO) RETOS program (RTC-2017-6467-2). LO, JA, MM, MSe, SR-P, MS-P, and LJG-G are employees of GoodGut. IE, NE-M, PB, MG, EC, and MT are employees of Elias–Boulanger who have received funding from RTC-2017 program. SD-A was an employee of Boehringer-Ingelheim who has received public and private funding from other funds. The funders had no role in study design, data collection and analysis, decision to publish or manuscript preparation.

## Conflict of Interest

SD-A is employed by Boehringer Ingelheim International GmbH. The remaining authors declare that the research was conducted in the absence of any commercial or financial relationships that could be construed as a potential conflict of interest.

## Publisher's Note

All claims expressed in this article are solely those of the authors and do not necessarily represent those of their affiliated organizations, or those of the publisher, the editors and the reviewers. Any product that may be evaluated in this article, or claim that may be made by its manufacturer, is not guaranteed or endorsed by the publisher.
