# Supplementary material for: In vitro Prebiotic Effect of Bread-Making Process in Inflammatory Bowel Disease Microbiome
Source: Front Microbiol. 2021 Oct 11;12:716307. doi: 10.3389/fmicb.2021.716307 (PMC8543021; doi:10.3389/fmicb.2021.716307)
Supplement: Supplementary file 1 [file Data_Sheet_1.pdf]

Supplementary Material

Table S1. Chemical composition of breads pre and post *in vitro* digestions.

Components	Ebindb		Eblfb1		Eblfb2	
	Pre-digestion	Post-digestion	Pre-digestion	Post-digestion	Pre-digestion	Post-digestion
**Proteins [a]**	10.12	1.26	8.12	1.13	9.09	1.39
**Dietary fiber [a]**	2.6	< 1.0	3.13	1.5	3.27	1.7
**Starch [a]**	NA	5.5	NA	5.8	NA	6.8
**SCFA [b]**						
**Acetic acid**	NA	173.12	NA	163.72	NA	153.42
**Propionic acid**	NA	42.77	NA	31.61	NA	29.78
**Isobutyric acid**	NA	ND	NA	ND	NA	ND
**Butyric acid**	NA	ND	NA	ND	NA	ND
**Isovaleric acid**	NA	14.05	NA	ND	NA	ND
**Valeric acid**	NA	17.69	NA	10.66	NA	9.82
**Hexanoic acid**	NA	45.96	NA	25.61	NA	26.34

[a] Values are grams per 100 grams of sample.

[b] Values are micrograms per liter of sample.

Table S2. Biomarkers abundances in fecal samples of 3 UC patients incubated with different bread treatments. Values are means ± standard deviation of abundances given as logarithm of total number of genomic units per stool gram analyzed in triplicate. Values within a row with different superscript letters are significantly different (*p*-value < 0.05).

**Bacterial Markers ***	Incubation control	Substrate control	Ebindb 1 g	Ebindb 2 g	Eblfb1 2 g	Eblfb1 2 g	Eblfb2 1 g	Eblfb2 2 g	Pectin 100 mg
**EUB**	10.48 ± 0.21^{a}	10.16 ± 0.22^{b}	10.71 ± 0.18^{ac}	10.80 ± 0.17^{c}	10.66 ± 0.22^{ac}	10.75 ± 0.15^{ac}	10.52 ± 0.15^{a}	10.62 ± 0.11^{a}	10.61 ± 0.37^{ac}
**ECO**	3.19 ± 0.71^{a}	3.34 ± 0.84^{a}	3.94 ± 1.12^{a}	4.56 ± 1.46^{b}	3.69 ± 0.43^{a}	3.66 ± 0.69^{a}	3.51 ± 0.87^{a}	3.53 ± 0.76^{a}	4.62 ± 1.68^{b}
**FPRA**	9.82 ± 0.55^{a}	8.91 ± 0.38^{bd}	9.05 ± 1.15^{cd}	8.43 ± 1.36^{bcd}	9.16 ± 0.31^{cd}	9.31 ± 0.47^{d}	8.88 ± 0.24^{b}	9.14 ± 0.52^{bcd}	8.77 ± 1.81^{a-d}
**PGHI**	7.52 ± 3.46^{a}	7.06 ± 3.12^{b}	7.39 ± 3.37^{ac}	6.72 ± 3.28^{bc}	7.35 ± 3.34^{ac}	7.01 ± 3.08^{b}	7.32 ± 3.31^{ac}	7.06 ± 3.13^{bc}	7.04 ± 3.56^{a}
**PGHII**	10.26 ± 0.32^{a}	9.25 ± 0.20^{b}	9.83 ± 0.41^{d}	9.60 ± 0.35^{cd}	9.60 ± 0.39^{cd}	9.44 ± 0.35^{bcd}	9.42 ± 0.54^{bcd}	9.23 ± 0.37^{bc}	10.39 ± 0.44^{a}
**ROS**	8.50 ± 0.16^{a}	6.95 ± 0.25^{b}	7.67 ± 0.46^{cd}	8.12 ± 0.65^{acd}	7.92 ± 0.65^{d}	8.23 ± 0.76^{ad}	7.48 ± 0.39^{c}	8.39 ± 0.50^{ad}	6.83 ± 0.40^{b}
**AKK**	4.21 ± 0.56	3.58 ± 0.47^{a}	3.97 ± 0.44	3.91 ± 0.31	4.65 ± 1.64^{b}	4.09 ± 0.39^{b}	4.01 ± 0.66	3.88 ± 0.37	3.65 ± 0.41^{a}
**B46**	7.97 ± 0.15^{a}	7.16 ± 0.15^{bd}	7.56 ± 0.29^{c}	7.22 ± 0.38^{b}	7.38 ± 0.26^{bc}	7.07 ± 0.23^{d}	7.24 ± 0.35^{bc}	6.98 ± 0.23^{d}	8.00 ± 0.48^{a}
**RUM**	8.97 ± 0.41	8.97 ± 0.37^{a}	9.31 ± 0.58^{a}	9.38 ± 0.78^{a}	9.29 ± 0.49^{a}	9.06 ± 0.65^{a}	9.12 ± 0.37^{a}	9.34 ± 0.84^{a}	8.54 ± 0.58^{b}
**LAC**	6.05 ± 1.94	5.64 ± 2.01	6.14 ± 1.94	6.72 ± 1.50	5.92 ± 1.71	6.50 ± 1.66	5.53 ± 1.83	6.00 ± 1.50	6.38 ± 1.37
**FIR**	10.53 ± 0.14^{a}	10.11 ± 0.09^{b}	10.44 ± 0.17^{a}	10.57 ± 0.18^{a}	10.35 ± 0.16^{ac}	10.54 ± 0.18^{a}	10.15 ± 0.28^{bc}	10.40 ± 0.15^{a}	10.52 ± 0.29^{a}
**BAC**	9.72 ± 0.19^{a}	9.73 ± 0.16^{a}	10.24 ± 0.24^{b}	10.35 ± 0.19^{b}	10.26 ± 0.25^{b}	10.22 ± 0.14^{b}	10.19 ± 0.17^{b}	10.16 ± 0.21^{b}	10.13 ± 0.33^{b}
**XIV**	8.78 ± 1.01^{ac}	7.46 ± 1.97^{b}	8.48 ± 0.96^{abc}	8.68 ± 1.56^{c}	8.36 ± 0.98^{abc}	8.80 ± 0.91^{ac}	8.27 ± 0.72^{abc}	8.03 ± 1.79^{abc}	8.21 ± 0.81^{ab}

* EUB, Eubacteria; ECO, *Escherichia coli*; FPRA, *F. prausnitzii*; PHGI, *F. prausnitzii phylogropup I*; PHGII, *F. prausnitzii phylogroup II*; ROS, *Roseburia spp.*; AKK, *A. muciniphila*; B46, best match BLAST *Subdoligranulum variabile*; RUM, *Ruminococcus spp.*; LAC, *Lactobacillus*; FIR, Firmicutes; BAC, Bacteroidetes; XIV, *Clostridial cluster XIV*.

Table S3. Biomarkers abundances in fecal samples of 3 CD patients incubated with different bread treatments. Values are means ± standard deviation of abundances given as logarithm of total number of genomic units per stool gram analyzed in triplicate. Values within a row with different superscript letters are significantly different (p-value < 0.05).

**Bacterial markers***	Incubation control	Substrate control	Ebindb 1 g	Ebindb 2 g	Eblfb1 2 g	Eblfb1 2 g	Eblfb2 1 g	Eblfb2 2 g	Pectin 100 mg
**EUB**	$10.49 \pm 0.35^{a-e}$	10.26 ± 0.39^{b}	10.49 ± 0.20^{acd}	10.79 ± 0.26^{ef}	10.48 ± 0.11^{c}	10.67 ± 0.15^{def}	10.44 ± 0.18^{ac}	10.63 ± 0.19^{adef}	10.81 ± 0.17^{f}
**ECO**	4.97 ± 0.70^{abc}	4.56 ± 0.75^{a}	4.75 ± 0.45^{ab}	5.21 ± 0.18^{c}	4.83 ± 0.44^{ab}	5.21 ± 0.17^{c}	4.73 ± 0.51^{ab}	5.11 ± 0.33^{ab}	5.00 ± 0.51^{ac}
**FPRA**	9.74 ± 0.48^{a}	9.27 ± 0.59	9.47 ± 0.43	9.73 ± 0.58	9.37 ± 0.41^{b}	9.48 ± 0.56	9.23 ± 0.40^{b}	9.49 ± 0.50	9.77 ± 0.74
**PGHI**	7.24 ± 2.99^{a}	7.13 ± 2.93^{ab}	7.03 ± 3.11^{ab}	7.00 ± 3.08^{ab}	6.71 ± 2.88^{b}	6.91 ± 3.01^{ab}	6.93 ± 2.79^{b}	6.81 ± 2.93^{b}	7.60 ± 3.24^{c}
**PGHII**	10.02 ± 0.57^{a}	9.46 ± 0.66^{b}	9.46 ± 0.56^{ab}	9.65 ± 0.69^{ab}	9.29 ± 0.41^{bc}	9.56 ± 0.70^{ab}	9.22 ± 0.41^{b}	9.49 ± 0.60^{bc}	9.94 ± 0.74^{ac}
**ROS**	8.24 ± 0.80^{acd}	7.50 ± 0.76^{be}	7.22 ± 0.53^{b}	8.19 ± 0.40^{c}	7.22 ± 0.77^{b}	8.64 ± 0.32^{d}	7.30 ± 0.91^{ab}	8.42 ± 0.76^{cd}	8.20 ± 0.85^{cde}
**AKK**	4.48 ± 2.42^{a}	4.33 ± 2.23	5.35 ± 2.10	5.67 ± 1.79^{b}	5.11 ± 2.04	5.27 ± 1.84	5.25 ± 2.21	5.17 ± 1.93	5.59 ± 1.78^{b}
**B46**	7.58 ± 0.50^{ad}	7.17 ± 0.56^{ab}	7.40 ± 0.45^{abd}	7.66 ± 0.58^{acd}	7.08 ± 0.23^{bc}	7.25 ± 0.56^{abd}	6.91 ± 0.26^{b}	7.27 ± 0.55^{abd}	7.72 ± 0.76^{cd}
**RUM**	8.92 ± 0.55	8.62 ± 0.37^{a}	9.13 ± 0.43	9.38 ± 0.83^{b}	9.07 ± 0.51	9.28 ± 0.69^{b}	9.01 ± 0.51	9.17 ± 0.61	9.16 ± 0.54
**LAC**	7.30 ± 1.53	7.14 ± 1.51	7.64 ± 1.31	7.81 ± 1.29	7.38 ± 1.07	7.62 ± 1.19	6.72 ± 1.97	7.42 ± 1.07	7.58 ± 1.24
**FIR**	10.53 ± 0.27^{abd}	10.29 ± 0.25^{bc}	10.42 ± 0.22^{ac}	10.68 ± 0.31^{ade}	10.32 ± 0.14^{c}	10.66 ± 0.20^{de}	10.39 ± 0.20^{ac}	10.60 ± 0.29^{ade}	10.76 ± 0.25^{e}
**BAC**	9.89 ± 0.55^{ab}	9.72 ± 0.48^{b}	10.02 ± 0.21^{acd}	10.27 ± 0.15^{e}	9.99 ± 0.11^{c}	10.17 ± 0.15^{de}	9.98 ± 0.23^{ac}	9.99 ± 0.33^{abd}	10.23 ± 0.12^{e}
**XIV**	8.62 ± 0.88	8.25 ± 0.94^{a}	8.50 ± 0.26^{a}	9.08 ± 0.55^{b}	8.45 ± 0.96^{a}	9.30 ± 0.47^{b}	8.02 ± 1.09^{a}	8.86 ± 0.75	9.25 ± 0.35^{b}

* EUB, Eubacteria; ECO, *Escherichia coli*; FPRA, *F. prausnitzii*; PHGI, *F. prausnitzii phylogropup I*; PHGII, *F. prausnitzii phylogroup II*; ROS, *Roseburia spp.*; AKK, *A. muciniphila*; B46, best match BLAST *Subdoligranulum variabile*; RUM, *Ruminococcus spp.*; LAC, *Lactobacillus*; FIR, Firmicutes; BAC, Bacteroidetes; XIV, *Clostridial cluster XIV.*

Table S4. Comparison of SFCA concentrations analyzed in fecal samples of UC patients among different treatments. Values are means ± standard deviation in micrograms per liter of three samples analyzed in triplicate (N = 9). Values within a row with different superscript letters are significantly different (*p*-value < 0.05).

SCFA	Substrate control	ebindb 1 g	ebindb 2 g	eblfb1 1 g	eblfb1 2 g	eblfb2 1 g	eblfb2 2 g	Pectin 100 mg
**Acetic acid**	489.98 ± 140.05[a]	1426.80 ± 399.96[bc]	1959.62 ± 527.45[d]	1388.67 ± 210.47[bc]	1912.90 ± 375.56[cd]	1258.84 ± 263.51[b]	1788.92 ± 450.78[bcd]	2556.24 ± 1477.47[d]
**Propionic acid**	353.47 ± 118.06[a]	1036.55 ± 370.21[b]	1593.37 ± 575.59[d]	1021.73 ± 212.26[b]	1478.48 ± 465.39[cd]	937.32 ± 210.76[be]	1373.98 ± 563.74[bc]	743.48 ± 251.11[e]
**Isobutyric acid**	65.01 ± 20.87	90.44 ± 58.98[a]	64.04 ± 48.37[b]	75.68 ± 32.42	72.08 ± 51.84	65.27 ± 17.64	57.32 ± 29.25[b]	71.44 ± 22.27
**Butyric acid**	270.14 ± 54.47[a]	1114.16 ± 743.50[bce]	1667.85 ± 648.51[de]	904.70 ± 371.74[b]	1887.9 ± 794.14[d]	907.25 ± 152.84[bc]	1392.72 ± 426.78[cd]	695.2 ± 194.56[b]
**Isovaleric acid**	115.75 ± 43.50[ac]	143.43 ± 109.47[ab]	99.36 ± 83.36[cd]	111.15 ± 58.00[abd]	115.86 ± 89.21[abd]	92.17 ± 33.56[bd]	86.2 ± 45.83[bd]	93.79 ± 47.03[bd]
**Valeric acid**	94.47 ± 51.39[a]	191.06 ± 118.90[bc]	206.28 ± 127.42[c]	180.02 ± 107.49[bc]	183.08 ± 138.19[bc]	156.81 ± 122.49[bd]	174.73 ± 109.50[bc]	136.23 ± 88.07[d]
**Hexanoic acid**	24.10 ± 34.69[a]	58.22 ± 87.14	59.52 ± 77.53[b]	56.99 ± 84.18	38.73 ± 69.37	58.51 ± 86.54	48.10 ± 69.42[b]	42.47 ± 57.23

Table S5. Comparison of SFCA concentrations analyzed in fecal samples of CD patients among different treatments. Values are means ± standard deviation in micrograms per liter of three CD samples analyzed in triplicate (N = 9). Values within a row with different superscript letters are significantly different (p-value < 0.05).

SCFA	Substrate control	Ebindb 1 g	Ebindb 2 g	Eblfb1 1 g	Eblfb1 2 g	Eblfb2 1 g	Eblfb2 2 g	Pectin 100 mg
**Acetic acid**	505.75 ± 127.58^{a}	1233.83 ± 251.25^{b}	1958.61 ± 307.89^{c}	1365.97 ± 219.94^{b}	1979.95 ± 794.72^{c}	1286.09 ± 180.43^{b}	2157.08 ± 436.36^{c}	2157.09 ± 436.36^{c}
**Propionic acid**	268.41 ± 121.81^{a}	729.29 ± 366.31^{b}	960.44 ± 621.04^{bc}	791.51 ± 475.41^{b}	1364.25 ± 563.71^{c}	816.54 ± 380.00^{b}	1308.12 ± 548.92^{c}	1308.12 ± 548.92^{b}
**Isobutyric acid**	55.90 ± 11.23^{a}	72.00 ± 21.50^{b}	177.86 ± 268.76^{c}	64.17 ± 10.78^{b}	69.01 ± 15.73^{b}	60.81 ± 12.92^{ab}	72.83 ± 16.62^{bc}	72.83 ± 16.62^{abc}
**Butyric acid**	270.79 ± 125.79^{a}	688.00 ± 371.99^{b}	1108.14 ± 500.98^{c}	729.38 ± 409.62^{b}	1228.04 ± 765.58^{c}	623.11 ± 337.46^{b}	1212.90 ± 621.22^{c}	1212.9 ± 621.22^{bc}
**Isovaleric acid**	99.27 ± 22.76^{ad}	116.45 ± 42.14^{bc}	140.24 ± 69.78^{c}	98.01 ± 20.14^{ab}	103.46 ± 28.86^{ab}	93.41 ± 25.41^{abd}	108.23 ± 33.64^{abc}	108.23 ± 33.64^{d}
**Valeric acid**	66.06 ± 22.48^{a}	115.22 ± 29.46^{b}	137.17 ± 53.03^{b}	121.67 ± 30.88^{b}	132.75 ± 85.82	119.06 ± 26.49^{b}	135.66 ± 76.59	135.65 ± 76.59^{a}
**Hexanoic acid**	4.72 ± 7.79^{a}	30.77 ± 46.09^{ab}	52.42 ± 76.31^{bc}	39.18 ± 57.52^{ab}	68.11 ± 93.68^{c}	40.59 ± 55.71^{ab}	59.88 ± 84.76^{bc}	177.16 ± 0.66^{d}

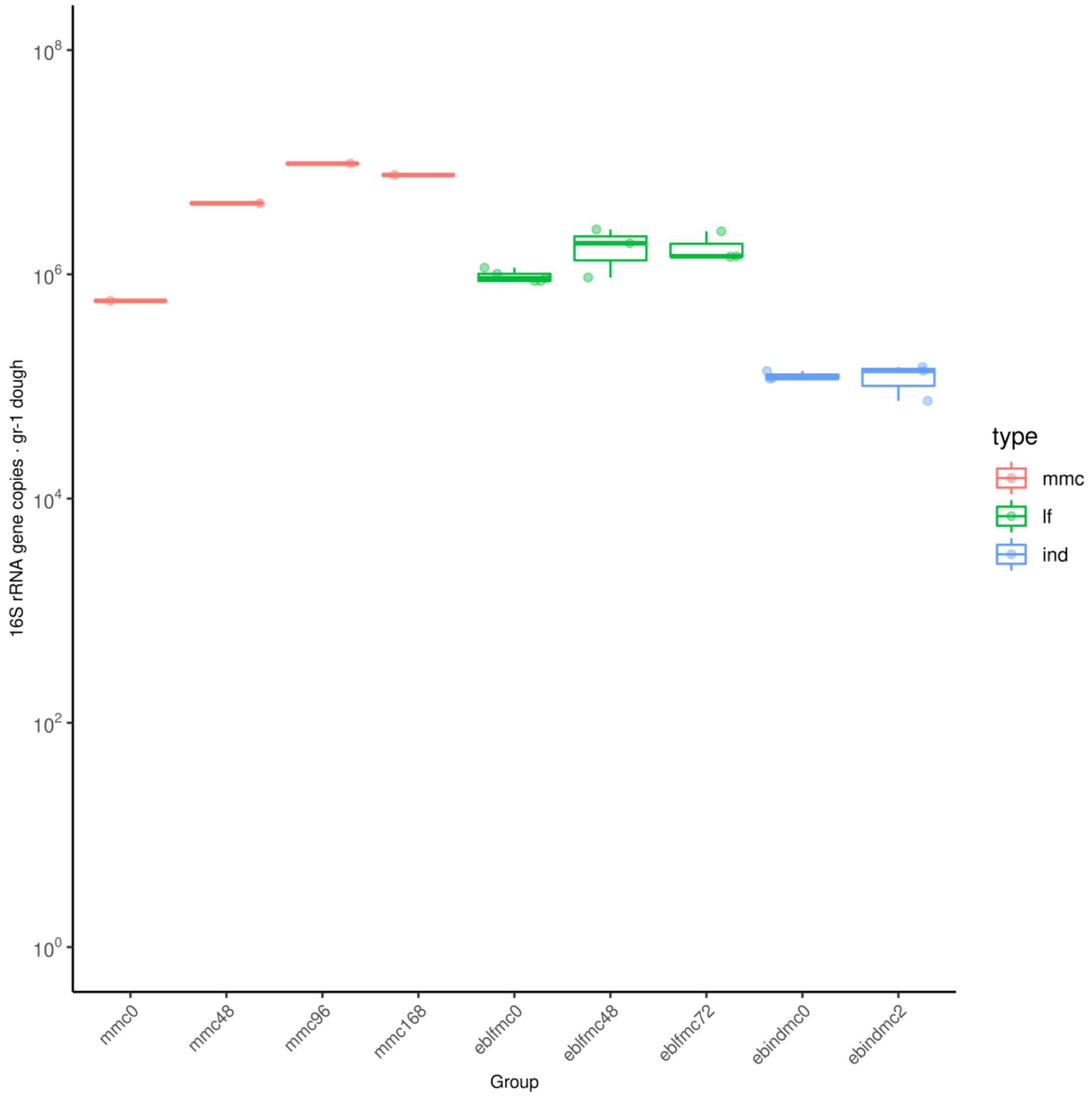

Figure S1. Representation of number of 16S rRNA gene copies per gram of the different types of bread doughs (MMC, sourdough; eblfmc, Elias-Boulanger long-fermentation raw dough; ebindmc, Elias-Boulanger industrial raw dough) at different times of fermentation.

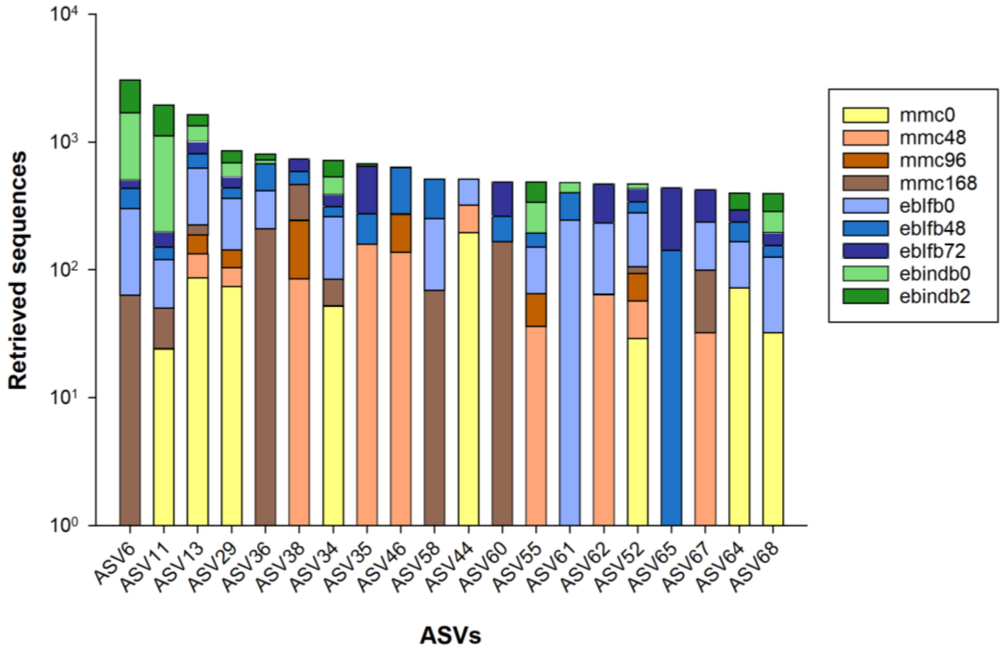

Figure S2. Rank abundance plot showing the top 20 most abundant retrieved ASVs and their abundance in samples grouped according to type of bread doughs and fermentation time for bacteria (16S rRNA gene).

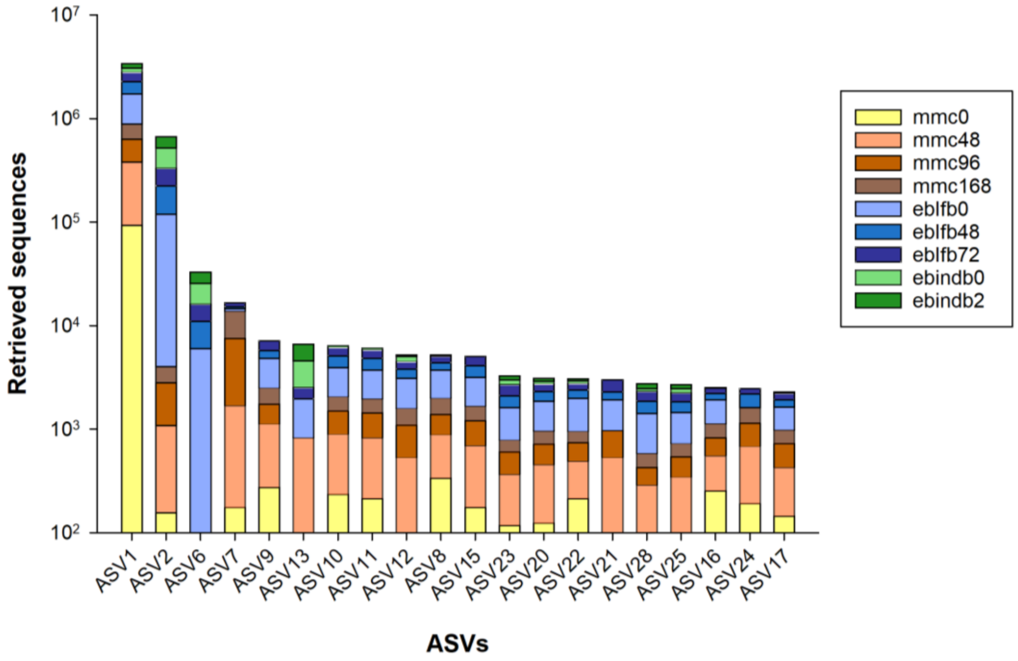

Figure S3. Rank abundance plot showing the top 20 most abundant retrieved ASVs and their abundance in samples grouped according to type of bread doughs and fermentation time for yeast (18S rRNA gene).

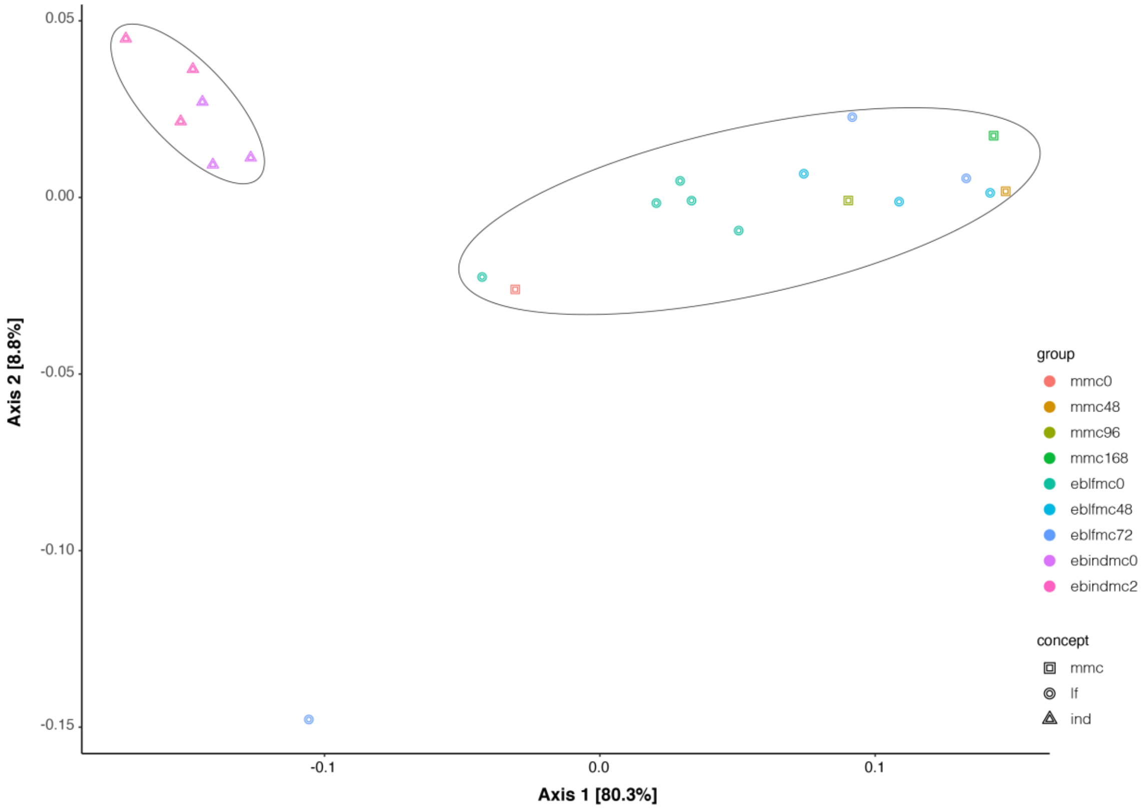

Figure S4. Principal coordinate analysis (PCOA) based on the balanced unifrac metric from the mass sequencing analyses of the different types of bread doughs (MMC, sourdough; eblfmc, Elias-Boulanger long-fermentation raw dough; ebindmc, Elias-Boulanger industrial raw dough) at different times of fermentation for bacteria (16S rRNA gene)

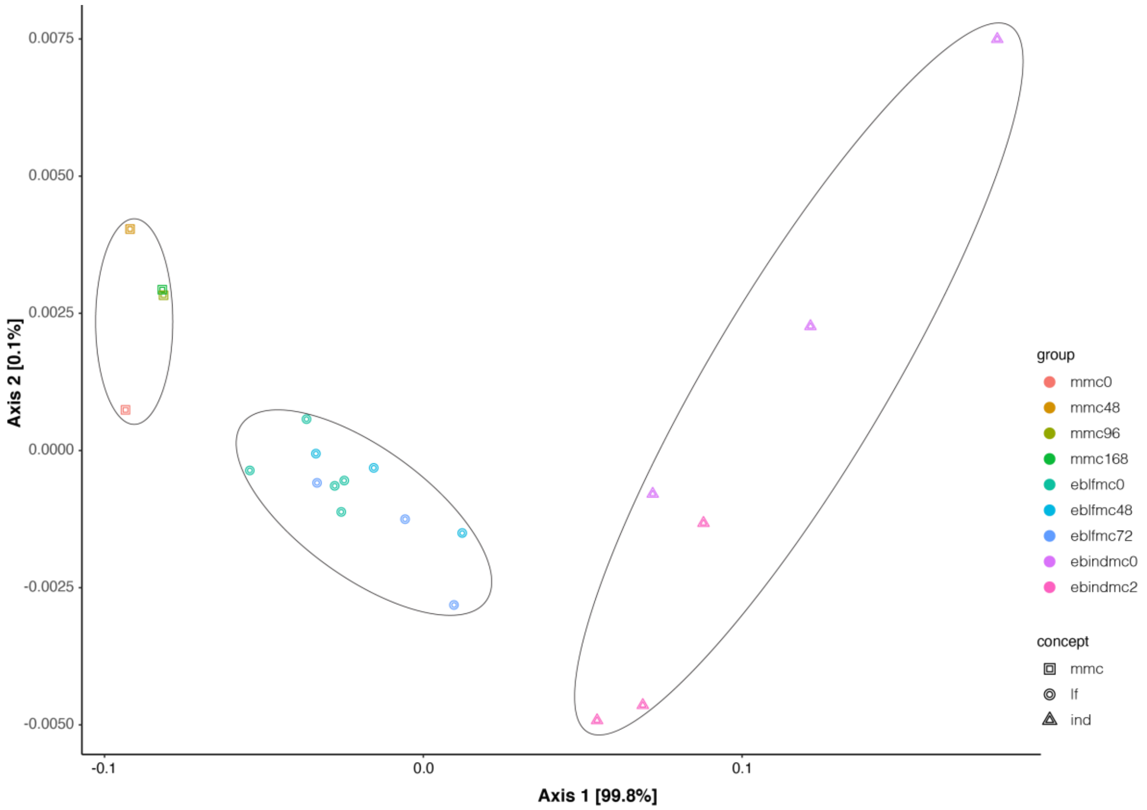

Figure S5. Principal coordinate analysis (PCOA) based on the balanced unifrac metric from the mass sequencing analyses of the different types of bread doughs (MMC, sourdough; eblfmc, Elias-Boulanger long-fermentation raw dough; ebindmc, Elias-Boulanger industrial raw dough) at different times of fermentation for yeast (18S rRNA gene).
